# Short- versus long-term outcomes after treatment for tuberculosis

**DOI:** 10.1186/1745-6215-14-S1-P44

**Published:** 2013-11-29

**Authors:** Laura Bonnett, Gerry Davies

**Affiliations:** 1 University of Liverpool, Liverpool, UK

## Background

TB remains a major killer amongst infectious diseases and current treatment involves a four-drug regimen for at least six months. Clinical development of a single novel TB drug is expected to take at least six years. A completely novel combination regimen would require twenty years or more. New drugs and regimens are required to shorten treatment duration, reduce toxicity and combat drug resistance.

We reviewed the ability of short-term outcomes from phase II trials to predict longer-term outcomes from phase III trials and hence improve selection of optimum combinations of new and existing drugs for development in pivotal trials.

## Methods

Phase II or phase III trials of combinations of eight agents for drug sensitive individuals with tuberculosis were included in our systematic review. Definitive clinical endpoints included treatment failure and treatment relapse. Early clinical endpoints incorporated positive or negative culture at various time points, time to sputum culture conversion and serial viable colony counts.

For categorical data, the odds ratio will be calculated using the Mantel-Haenszel method, and for continuous data, such as colony counts, the mean difference will be calculated. Time to event outcomes will be summarised via the generic inverse-variance method. Additionally, early endpoints will be evaluated as surrogate outcomes for poor outcome via the generalised R2 statistic.

## Results

2865 trials were identified for potential inclusion in the review. Of these, 47 phase II and 478 phase III trials were included. Data is currently being extracted and full results will be presented.

